# Prevalence and resistance pattern of uropathogens from community settings of different regions: an experience from India

**DOI:** 10.1099/acmi.0.000321

**Published:** 2022-02-09

**Authors:** Sarita Mohapatra, Rajashree Panigrahy, Vibhor Tak, Shwetha J. V., Sneha K. C., Susmita Chaudhuri, Swati Pundir, Deepak Kocher, Hitender Gautam, Seema Sood, Bimal Kumar Das, Arti Kapil, Pankaj Hari, Arvind Kumar, Rajesh Kumari, Mani Kalaivani, Ambica R., Harshal Ramesh Salve, Sumit Malhotra, Shashi Kant

**Affiliations:** ^1^​ Department of Microbiology, All India Institute of Medical Sciences, New Delhi, India; ^2^​ Department of Microbiology, SUM Hospital, Bhubaneswar, India; ^3^​ Department of Microbiology, All India Institute of Medical Sciences, Jodhpur, India; ^4^​ Department of Microbiology, Bangalore Medical College and Research Institute, Karnataka, India; ^5^​ Translational Health Science and Technology Institute, Faridabad, India; ^6^​ Department of Pediatrics, All India Institute of Medical Sciences, New Delhi, India; ^7^​ Department of Medicine, All India Institute of Medical Sciences, New Delhi, India; ^8^​ Department of Obstetrics & Gynaecology, All India Institute of Medical Sciences, New Delhi, India; ^9^​ Department of Biostatistics, All India Institute of Medical Sciences, New Delhi, India; ^10^​ Centre for Community Medicine, All India Institute of Medical Sciences, New Delhi, India

**Keywords:** urinary tract infection, antimicrobial resistance, community-acquired urinary tract infection, India, uropathogenic *E. coli*, *Klebsiella pneumoniae*

## Abstract

**Introduction:**

Urinary tract infection (UTI) is one of the most common infections in clinical practice worldwide in both healthcare and community settings causing significant morbidity and mortality. It is one of the major conditions at the community level treated empirically and regarded as a potential cause of emergence of antimicrobial resistance (AMR). Limited information is available regarding community-acquired UTI (CA-UTI) from India.

**Methodology:**

This is a first of its kind, multicentric-cross-sectional study at the community level targeting patients attending the out-patient department (OPD) of the community health centre (CHC) from four geographical regions (North, South, West and East) of India. The study had been designed to determine the epidemiology, antibiogram profile and identification of extended-spectrum beta-lactamase (ESBL) producer and carbapenem resistant (CR) uropathogens. Samples were collected prospectively from UTI suspected patients coming at CHC and processed at the tertiary healthcare centres using a common standard operating procedure. Clinical history of all the patients exhibiting significant bacteriuria was collected and data was analysed.

**Result:**

Overall, 250 out of a total of 2459 (10.1 %) urine samples were positive for bacteria with significant bacteriuria (adult: paediatrics, 6.7 : 1). Females were predominantly affected (male: female, 1 : 2.9). History of recent episode of UTI was observed as the commonest risk factor followed by diabetes mellitus. Altogether, 86 % of total cases were caused by *Escherichia coli* (68 %) and *Klebsiella pneumoniae* (17.6 %) together. Among the commonly used oral antibiotics for the Gram-negative bacilli (GNB), the highest resistance was observed against beta-lactams, first- and second-generation cephalosporins, fluoroquinolones and co-trimoxazole. Overall, the prevalence of ESBL producer and CR isolates were 44.8, and 4.3 %, respectively. However, the ESBL production, CR and nitrofurantoin resistance among the uropathogenic *E. coli* (UPEC) isolates was 52.8, 5.1 and 14 %, respectively. No resistance was found against fosfomycin among the UPEC isolates.

**Conclusion:**

The current study highlights the increasing incidence of AMR among uropathogens at the community-settings of India. A significant percentage of ESBL producers among the isolated UPEC and *K. pneumoniae* were observed. The currently available evidence supports the clinical recommendation of fosfomycin and nitrofurantoin for empiric therapy in CA-UTI in India.

## Introduction

Urinary tract infection (UTI) is one of the most common infectious diseases worldwide. It is more prevalent among females with an incidence rate 50-fold higher among the 20–50 years age group. There are various clinical manifestations of UTI such as cystitis, pyelonephritis, asymptomatic bacteriuria, chronic and recurrent UTIs; of which cystitis is the most frequent presentation affecting the urinary bladder [1]. The bacteria may further ascend in the urinary tract causing infection of the kidney; if timely management is not done [2]. Majority cases of uncomplicated UTI are community-acquired in origin and caused by uropathogenic *E. coli* (UPEC) and *Klebsiella* spp., constituting approximately 75–95 % of the total cases [3]. The other less prevalent organisms are *Proteus* spp., *Enterobacter* spp., *Pseudomonas* spp., *Enterococcus faecalis *, *Staphylococcus saprophyticus* and *Staphylococcus aureus* [4]. Diagnosis is made based on the presenting symptoms and significant bacteriuria, i.e. ≥10^5^ colony forming units of organism on culture [5]. The outcome of UTI may also depend upon the presence of underlying host factors such as age, diabetes, spinal cord injury and urinary catheterization among various others [6, 7].

Extended-spectrum beta-lactamase (ESBL) producing *E. coli* and *K. pneumoniae* causing CA-UTI has been increasingly reported from many parts of the world including India and are co-resistant to other non-beta-lactam antibiotics [8]. Moreover, the recent emergence of multidrug-resistant (MDR) UPEC isolates in the community have become a major challenge for the clinician to start the empirical therapy. Community-acquired UTI (CA-UTI) makes up a large proportion of infections attending out-patient departments (OPDs) of hospitals and a substantial amount of oral antibiotics are being prescribed for the treatment. Hence, it is extremely imperative to determine the common aetiology based on the local epidemiology and the antibiotic susceptibility testing (AST) pattern to formulate the treatment prescription for empirical therapy. Although, there are many studies describing the antimicrobial resistance (AMR) pattern; but limited data is available on the local prevalence of uropathogens among the CA-UTI group patients [9, 10]. Thus, we conducted a prospective multicentric study at community health centres (CHCs) from different geographical regions of India to determine the aetiology, prevalence with underlying risk factors and its AMR pattern.

## Methodology

This was a prospective multicentric cross-sectional study conducted at the community level targeting patients attending the OPD of the CHCs from four different geographical regions of India over a period of 1 year.

The centres included in the study were as follows: centre 1 (New Delhi), centre 2 (Bangalore), centre 3 (Bhubaneswar) and centre 4 (Jodhpur). Urine samples from suspected UTI patients were collected at CHC and processed at the tertiary health centres using the common standard operating procedure.

Criteria for sample collection, transport and procedure were defined as follows:

- *Case definition*: consecutive patients with age above 1.5 years from the defined community presenting to OPD with any of the following symptoms as chief complaints were included in this study.
- *Inclusion criteria*: uncomplicated UTI (without any anatomical or functional abnormality), cystitis, burning micturition, increase in frequency, urgency, pain above pubic symphysis, asymptomatic bacteriuria in pregnant women, recurrent UTI and unexplained fever.
- *Exclusion criteria*: asymptomatic bacteriuria in conditions other than pregnancy, history of hospitalization (1 week prior to presentation, or more than 48 h), known case of neurogenic bladder, obstructive uropathy, pediatric patients with vesicoureteral reflex and on antibiotic prophylaxis.

### Sample collection

Clean catch mid-stream urine sample was collected from the patients and transported to the referral tertiary care health centres for further processing.

### Identification

The urine samples were cultured on cysteine lactose electrolyte deficient media and incubated at 35±2 °C overnight. Colony count of a single pure organism with ≥10^3^ (c.f.u. ml^-1^) was regarded as significant bacteriuria [11]. Identification to species level was done for all isolates using VITEK-2 (Biomeriux, Durhum, USA) or matrix-assisted laser desorption ionization time-of-flight mass spectrometry (MALDI-TOF MS) (VITEK MS, BioMerieux, France). For identification using MALDI-TOF MS, a single pure colony of the bacterium was directly spotted onto the MALDI-TOF MS sample slide. Matrix α-cyano-4-hydroxycinnamic acid was added to the sample and allowed to air dry for 5 mins. The slide was then inserted into the MALDI-TOF MS machine for laser desorption/ionization. The mass spectrum data was then compared with reference database to identify the bacteria [12].

### Antibiotic susceptibility testing

AST of the isolates was performed by VITEK-2 (Biomeriux, Durhum, USA) using Gram-negative AST Cards, with strict adherence to the Clinical Laboratory Standard Institute 2020 guidelines. Antibiotics tested for Gram-negative bacilli (GNB) include the following: ampicillin, amoxicillin/clavulanic acid, piperacillin/tazobactam, cefalotin, cefoxitin, cefixime, ceftazidime, ceftriaxone, ertapenem, amikacin, gentamicin, ciprofloxacin, norfloxacin, fosfomycin, nitrofurantoin and co-trimoxazole; while antibiotics tested for Gram-positive cocci (GPC) were benzylpenicillin, gentamicin (high-level), ciprofloxacin, levofloxacin, erythromycin, linezolid, teicoplanin, vancomycin, tetracycline and tigecycline [13, 14].

## Results

*Demographic data*: A total of 2459 urine samples were processed from all four centres of which 10.1 % (*n*=250) were positive for uropathogens showing significant bacteriuria. Females were predominantly infected (72.5 %), followed by males (27.5 %) (Table 1). Overall, 43.1 % (769/1781) of total female patients were found pregnant. The distribution of adults and pediatrics patients were observed as 89.2% and 10.8 %, respectively. Among the adults, the highest prevalence was observed in the 19–35 years (56.9%) age group, followed by 36–55 years (19.4 %), and 56–100 years (12.5 %). 67.3 % of the patients were married and 14.5 % were unmarried. The total number of paediatric patients was 320 of which 44.4 % were below 10 years of age while 55.6 % were above 10 years of age. Based on occupation, majority were identified as housewives (44.4 %), followed by employees (26.8 %), students (13.3 %), farmers (4.8 %) and drivers (1.5 %).

**Table 1.** Demographic data of patients from different centres

	Centre 1 (North India)	Centre 2 (South India)	Centre 3 (East India)	Centre 4 (West India)	Total no. and (%)
Total sample	1043	452	825	139	2459
Total positive sample	55	51	104	40	250
Male	245	34	343	56	678 (27.5 %)
Female	798	418	482	83	1781 (72.5 %)
Age					
(18-35) (35-55) (>55)	678 161 72	368 20 4	278 265 195	77 33 38	1401 (56.9 %,) 479 (19.4 %) 309 (12.5 %)
Occupation					
Students Employee Housewife Driver Farmers	190 72 650 17 26	44 281 274 0 2	78 236 208 6 19	12 71 0 3 72	324 (13.3 %) 660 (26.8 %) 1132 (44.4 %) 26 (1.5 %) 119 (4.8 %)
Married	859	388	280	130	1657 (67.3 %)
Unmarried	223	59	47	28	357 (14.5 %)
Education					
Primary Secondary Higher	434 499 109	58 101 230	31 179 287	97 42 19	620 (25.2 %) 821 (33.3 %) 645 (26.2 %)
Pregnancy	265	365	9	18	769 (43.17 %)*

*Calculated out of the total number of females.

In the current study, burning micturition was observed as the commonest symptom (37.6 %) followed by frequency (30.4 %), urgency (29.1 %), suprapubic pain (23 %) and dribbling of urine (6.5 %) . Flank pain (27.8 %) was observed as the commonest complaint by the patients followed by turbid/cloudy urine (23 %), fever (20.8 %), malodorous urine (11.3 %), fever with loin pain (13.6 %) and hematuria (3.7 %). Among the co-morbidities, patients had a history of recent episode of UTI (23.9 %), diabetes mellitus (3.2 %), renal stone (1.1 %), sexually transmitted diseases (0.3 %), bladder atony (1.01 %), urological surgery (0.1 %) and recurrent urinary tract infection (0.4 %).

### Microbiological data

A total of 250 samples were positive for uropathogens (10.1 %) out of 2459 urine specimens processed in the microbiology labs. GNB constituted 94.4 % and GPC constituted 5.6 % of the total isolates. The prevalence of CA-UTI infections was ranged between 5–13 % for three centres (centres 1, 2 and 3) except for centre 4, where the prevalence was observed to be around 28.7%.

*E. coli* was observed as the commonest uropathogen (68 .3%), followed by *K. pneumoniae* (17.6 %), *Proteus* spp. (3.2 %), *Acinetobacter* spp. (1.2%), *Enterococcus* spp. (5.6 %) and others (* K. oxytoca *, *Enterobacter cloacae, Pseudomonas aeruginosa, Serratia marcescens, Providencia rettgeri, Citrobacter fruendii* and *Citrobacter koseri *) (4 %). [Table 2] *E *. *coli* and *K. pneumoniae* uniformly constituted the major part of uropathogens in all four centres.

**Table 2.** Distribution of uropathogen among the different centres

Centres	Total samples	Significant bacteriuria	* E. coli *	* K. pneumoniae *	* Proteus* spp.	* Acinetobacter* spp.	* Enterococcus* spp.	Others
Centre 1 (North India)	1043	55 (5.3 %)	41	2	3	1	7	1
Centre 2 (South India)	452	51 (11.3 %)	34	16	1	0	0	0
Centre 3 (East India)	825	104 (12.6 %)	65	22	3	2	2	10
Centre 4 (West India)	139	40 (28.7 %)	30	4	1	0	5	0
Total	2459	250 (10.1 %)	170 (68.3 %)	44 (17.7 %)	8 (3.2 %)	3 (1.2 %)	14 (5.6 %)	10 (4 %)

In the present study, the AMR pattern among the GNBs against commonly prescribed drugs for CA-UTI were found in the following manner [Table 3]: ampicillin (51.8 %), ticarcillin (50.2 %), amoxicillin-clavulanic acid (25.3 %), tazobactam-piperacillin (24.9 %), cefalotin (44.6 %), cefoxitin (22.1 %), cefixime (42.6 %), ceftriaxone (40.9 %) ceftazidime (31.7 %), ciprofloxacin (45.4 %), ofloxacin (37.8 %), norfloxacin (26.1 %), amikacin (7.2 %), gentamicin (14 %), ertapenem (4.4 %), co-trimoxazole (37.3 %) nalidixic acid (51.4 %), nitrofurantoin (13.3 %). No resistance was observed against fosfomycin by any of the UPEC isolates. Overall, the prevalence of ESBL producers and carbapenem-resistant organisms were 44.8%, and 4.3 %, respectively.

**Table 3.** Overall percentage of resistance to common antibiotics among Gram-negative bacilli uropathogens

Name of the antibiotic	Resistance rate (%)
Amikacin	7.2
Amoxycillin-clavulanic acid	25.3
Ampicillin	51.8
Cefalotin	44.6
Cefoxitin	22.1
Ceftazidime	31.7
Ceftriaxone	41.0
Ciprofloxacin	45.4
Co-trimoxazole	37.3
Ertapenem	4.4
Fosfomycin	0.0
Gentamicin	14.1
Nalidixic acid	51.4
Nitrofurantoin	13.3
Norfloxacin	26.1
Ofloxacin	37.8
Piperacillin-tazobactam	24.9
Ticarcillin	50.2
Cefixime	42.6

**Fig. 1.**
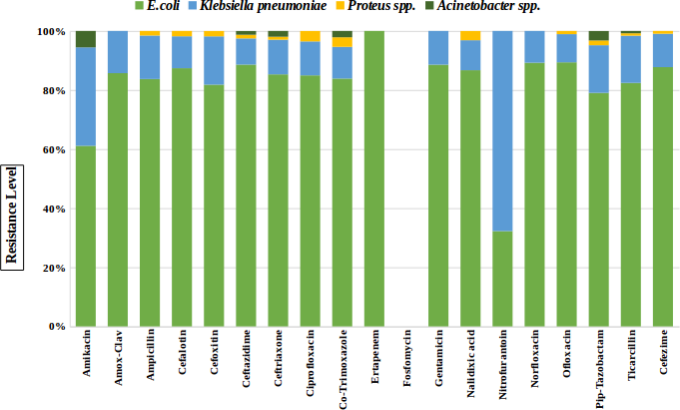
Bar diagram representation of common Gram-negative bacilli resistant to various antibiotics causing UTIs.

The distribution of AMR against the common GNB uropathogens (* E. coli *, *K.pneumoniae*, *Proteus* spp. and *Acinetobacter* spp.) to various antibiotics was represented in Fig. 1. *E. coli* was the predominant uropathogen and was observed to be resistant to most of the antibiotics in comparison to the rest of the uropathogens. However, the resistance against nitrofurantoin was dominated in the *K. pneumoniae* isolates in comparison to UPEC isolates.

**Fig. 2.**
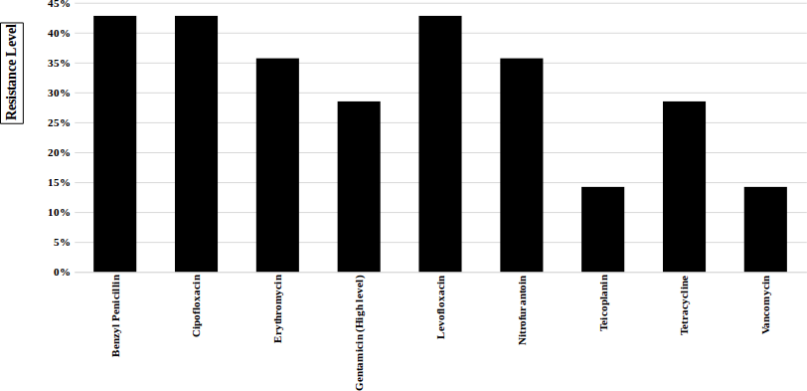
Bar diagram representation of *Enterococcus* spp. resistant to various antibiotics causing UTIs.

Resistance rates among the *Enterococcus* spp. was observed as follows (Fig. 2): benzylpenicillin (42.8 %), tetracycline (28.5 %), erythromycin (35.7 %), ciprofloxacin (42.8 %), levofloxacin (42.8 %), high-level gentamicin (28.5 %), nitrofurantoin (35.7 %), vancomycin (14.2 %), teicoplanin (14.2 %). No resistance was observed against linezolid.

**Fig. 3.**
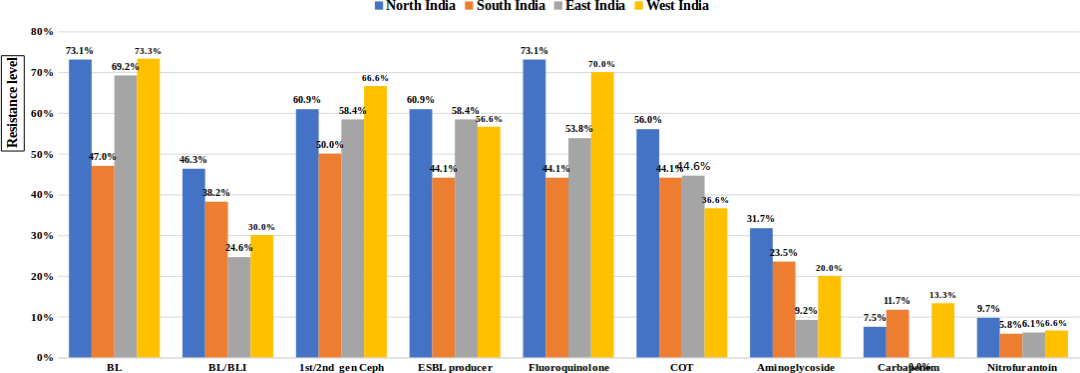
Bar diagram showing antimicrobial resistance pattern of uropathogenic *E. coli* (UPEC) isolates from different centres. BL – beta-lactams, BL/BLI – beta-lactams/beta-lactam Inhibitor combination, first/second generation ceph – first or second generation cephalosporins, COT – co-trimoxazole, ESBL producer – extended-spectrum beta-lactamase producer.

The AMR pattern of UPEC isolates against various antibiotics from different centres was described in Fig. 3. The resistance against beta-lactams, cephalosporins and fluoroquinolones, were observed approximately 50 % in all four centres. The resistance against co-trimoxazole and beta-lactam-beta-lactamase inhibitors were approximately ranged between 30–40 %. The resistance against aminoglycosides, carbapenems and nitrofurantoin was found between 9.2–31.7 %, 0–13% and 5.8–9.7%, respectively.

**Fig. 4.**
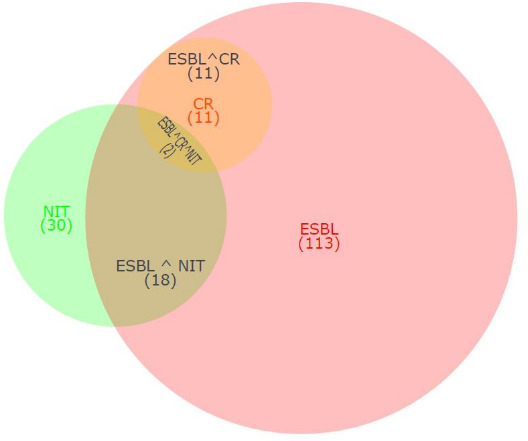
Venn diagram showing the relationship between ESBL positive, Carbapenem-resistant and nitrofurantoin-resistant isolates of *E. coli* and *K. pneumoniae *. ESBL– extended-spectrum beta-lactamase positive, CR – carbapenem resistant, NIT – nitrofurantoin resistant.

*E. coli* and *K. pneumoniae* belonging to the family *Enterobacteriaceae* together constituted 85.6 % (214/250) of the total uropathogens in the current study. On analysing their combined AMR data, it was observed that 52.8 % of the isolates in this group were ESBL producers, 5.14 % were carbapenem resistant and 14 % were resistant to nitrofurantoin (Fig. 4). All the isolates resistant to carbapenem were found to be a subset of ESBL producers indicating possible emergence of carbapenem resistance within the ESBL producers. Overall, 60 % (18/30) of the total nitrofurantoin resistant isolates were also observed positive for ESBL production. In total, 1.6 % of isolates were found to be MDR exhibiting resistance against nitrofurantoin, third-generation cephalosporin and carbapenem.

## Discussion

UTI is a common bacterial infection occurring in a large number of patients and women are particularly at greater risk of developing a UTI than men. We conducted a prospective study over a period of 1 year to determine the prevalence and antimicrobial resistance pattern of different uropathogens causing community-acquired UTI from different geographical regions of India.

A total of 250 uropathogens had been isolated and identified from urine specimens attending the OPD of the CHC from four different geographical regions of India representing the local epidemiology of CA-UTI at different places. In our current study, females were observed to be affected commonly with predominance during the pregnancy period and the study result is concordant to other studies reported from the neighbouring countries of the same geographical area [15]. The prevalence rate of CA-UTI in our study was found 10.1 % with a rate of 12.8 % (32/250) among paediatrics and 86.8 % (217/250) among adults. More than 85 % of the uropathogens belonged to *Enterobacterales* predominantly constituting *E. coli* and *K. pneumoniae *. Previous episodes of UTI and diabetes mellitus were seen to be more associated with the causation of infection in our patient group. It has been hypothesized that increased glucose levels may lead to the proliferation of bacteria among diabetic patients causing UTI [6, 7]. Hence, timely diagnosis with proper management is necessary to prevent any further complications due to UTI. Currently, an increasing incidence of MDR bacteria causing UTI had been reported from both hospital and community settings [16]. This increasing drug resistance was observed highest among the *E. coli* isolates.

There is a scarcity of literature available from India exhibiting the epidemiology of CA-UTI and the majority were focused on single centres [5]. In the latest studies from India [17–19], *E. coli* was reported as the predominant isolate (72 %) followed by *Klebsiella* spp. (15 %), which is concordant with our study results. Another study targeting the community setting from two different geographical parts of India reported *E. coli* as the most predominant isolate (55.1 %), followed by *E. faecalis* (15.8 %) and *K. pneumoniae* (13.7 %). However, *K. pneumoniae* was the second most common uropathogen after *E. coli* from northern India. The prevalence of *P. aeruginosa *, which is considered mostly a pathogen in nosocomial settings was 4.5 % and our study data also found its prevalence to be very low at 3.1 % [20]. In the present study, the resistance of GNB against the common oral antibiotics, i.e. co-trimoxazole, fluoroquinolones and cephalosporins (first and second generation) were 41.3, 50.2 and 49.3 %, respectively. As per the published literature, 10−15 % resistance against trimethoprim/sulfamethoxazole was observed from Asia and 30 % was observed from China and South Korea [21–23]. On comparing the fluoroquinolone susceptibility among the UPEC isolates, 90% were observed susceptible from Japan and Australia while 70–80% from the USA and 74–84 % from China [24]. Another meta-analysis from India targeting community-acquired and hospital-acquired UTIs also revealed a higher rate of fluoroquinolone resistance (>60 %) among the UPEC isolates [25]. In our study also, a high level of resistance to ciprofloxacin was observed especially in *E. coli* (84.9 %), showing concordance with the previous increasing resistance trend and setting up alarm in the sense that ciprofloxacin might become obsolete in empirical therapy for UTI cases in India.

The overall ESBL resistance among the GNBs in our study was 48 % and among the UPEC and *K. pneumoniae* isolates was 55 and 31.81%, respectively. The ESBL positivity rate among the UPEC isolates from different settings of India varied from 16.6–69.2 %. Moreover, the ESBL positivity among *K. pneumoniae* isolates of the same cohort was found to be more than 50 % [19]. These studies indicate the scattered and isolated nature of CA-UTI studies previously done in India. The varied ESBL prevalence rate may be attributed to the study population targeted at single centres at different geographical areas reflecting the local epidemiology of that area. Thus, epidemiological study of CA-UTI at the national level is a much-needed concern.

The current study is first of its kind targeting multiple community centres at different geographical areas in India with strict adherence to the common SOPs. Hence, this data will be helpful for the prediction of the actual prevalence rate of UTI at the community level and can be used as a reference backbone data for the formulation of empirical therapy for CA-UTI cases.

In our current study, the average rate of ESBL in *E. coli* and *K. pneumoniae* were 56.2 and 37.9 %, respectively. A recent study from China on CA-UTI found a lower rate (38 %) of ESBL among UPEC isolates. Comparing the susceptibility to carbapenems was observed marginally higher [imipenem (99.9 %), ertapenem (98.9 %)] in comparison to our study; where it was 96 % against ertapenem [26]. These results highlight the alarming rise of ESBL in the community settings across the world, which needs regular and stringent monitoring protocols to formulate the treatment guidelines, implementation of infection control measures for further reduction of its spread in the community [21, 27].

According to the European Association of Urology guidelines, nitrofurantoin is recommended for the treatment of uncomplicated cystitis as first-line empiric therapy [28]. In the current study, resistance to nitrofurantoin for UPEC isolates was 5.8 % and *K. pneumoniae* was 45.4 %. None of UPEC isolates were found resistant to fosfomycin. These results favour the use of nitrofurantoin and fosfomycin as a first-line antibacterial agent for the treatment of CA-UTI. The rates of resistance of UPEC to nitrofurantoin from different European countries were reported below 1.5 % [28]. Nitrofurantoin resistance in our study was observed higher for community-acquired UTI patients. Hence, judicious usage is warranted to prevent any further emergence of resistance against these two antibiotics.

## Conclusion

In conclusion, this multicentric study highlights increase in the incidence of AMR in uropathogens causing CA-UTI in India in comparison to earlier community studies conducted at single centres. Significant percentage of ESBL producers among the isolated UPEC and *K. pneumoniae* were observed. More attention to the choice of empirical antibiotic therapy is needed. The high frequency of uncomplicated bacterial CA-UTI in adults and their treatment with antibiotics exhibits high antibiotic selection pressure on the pathogen and also on the collateral flora, resulting in the selection of antibiotic-resistant bacteria in the population. Local sentinel surveillance for pathogens and their antimicrobial resistance rates in a given area with routine testing for ESBL producers is recommended to determine appropriate guidelines for CA-UTI management. This will further help in decreasing the improper use of antibiotics and the emergence of AMR in society.

Our study indicated that the use of cephalosporins and fluoroquinolone needs to be restricted for empirical treatment of CA-UTIs in India due to high resistance rates. Carbapenems can be preserved for highly suspected ESBL-producing and MDR strains. However, the occurrence of carbapenem resistance in the community is something that should not go ignored. The currently available evidence supports the clinical recommendation of fosfomycin and nitrofurantoin for empiric therapy in CA-UTI.
